# Development of a school-based digitalised intervention for ADHD using Intervention Mapping

**DOI:** 10.1192/j.eurpsy.2023.355

**Published:** 2023-07-19

**Authors:** A. Russell

**Affiliations:** University of Exeter Medical School, University of Exeter, Exeter, United Kingdom

## Abstract

**Introduction:**

Attention deficit/hyperactivity disorder (ADHD) is a prevalent and impairing neurodevelopmental disorder affecting 2-5% of children. These children are at risk of negative health, social and educational outcomes; ADHD incurs an estimated £670 million annual cost to health, education and social care in the UK. Children with ADHD often experience severe difficulties at school despite drug treatment: effective psychosocial interventions are needed. There is mixed evidence for the effectiveness of existing school-based interventions for ADHD, which are complex and resource-intensive, contradicting the preferences of teachers for short, flexible strategies that suit a range of ADHD-related classroom-based problems.

**Objectives:**

To develop a prototype of a school-based intervention for ADHD.

**Methods:**

Intervention Mapping, a framework for developing theory- and evidence-informed interventions with explicit consideration of implementation context, was used. Logic models were developed of the behaviour change steps required by each agent in the school system to improve outcomes for students with ADHD.

A comprehensive evidence synthesis was conducted for interventions that targeted the key outcomes of relevance (inattention, impulsivity, hyperactivity, peer and teacher relationships, self-esteem, executive functions and organisation skills); findings were integrated alongside behaviour change theory and theories of the underlying aetiology of ADHD, in order to develop a logic model for the intervention. Components of the intervention were then developed in line with the logic model using evidence-based behaviour change methods, with input from people with ADHD, school staff and other key stakeholders at every stage of the development process.

**Results:**

The development process resulted in a prototype digital platform that can be utilised to deliver a personalised behavioural intervention for children with ADHD within primary schools. It contains some core components that all teachers and children will complete, and then is individualised based on the key problems each child is currently facing. There are six optional modules, each containing a range of behavioural strategies for teachers to implement with the student or the whole class. The toolkit includes a symptom tracking graph that teachers can use to visualise the progress a child is making, and is developed to align with the current resources and capacity of primary schools in the UK.

**Conclusions:**

The prototype intervention is designed explicitly to fit with existing school structures and demands, and to be low cost in terms of delivery and training. It focusses on adapting the school environment to better suit children with high levels of ADHD symptoms. It is now being feasibility tested, and in this talk I will describe the development process using Intervention Mapping, and the initial feedback from the first testing of implementation of the prototype.

**Disclosure of Interest:**

None Declared

